# 血清miR-4646-5p、miR-3654联合传统肺癌肿瘤标志物在云南宣威肺癌诊断中的应用研究

**DOI:** 10.3779/j.issn.1009-3419.2024.101.23

**Published:** 2024-09-20

**Authors:** Renning ZHANG, Xinrui WAN, Xuan HUANG, Mingping LI, Kai XU, Raohong FANG, Ya LI

**Affiliations:** ^1^661100 蒙自，云南省滇南中心医院（红河州第一人民医院）检验科; ^1^Department of Clinical Laboratory Medicine, Yunnan Diannan Central Hospital (The First People's Hospital of Honghezhou), Mengzi 661100, China; ^2^650301 昆明，昆明市第三人民医院检验科; ^2^Department of Clinical Laboratory Medicine, The Third People's Hospital of Kunming, Kunming 650301, China; ^3^650032 昆明，昆明医科大学第一附属医院医学检验科; ^3^Department of Clinical Laboratory Medicine, The First Affiliated Hospital of Kunming Medical University, Kunming 650032, China; ^4^650032 昆明，云南省检验医学重点实验室; ^4^Yunnan Key Laboratory of Laboratory Medicine, Kunming 650032, China; ^5^650032 昆明，云南省医学检验临床医学研究中心; ^5^Yunnan Province Clinical Research Center for Laboratory Medicine, Kunming 650032, China

**Keywords:** 微小RNA, 宣威肺腺癌, 肺肿瘤, 肿瘤标志物, 诊断效能, microRNA, Xuanwei lung adenocarcinoma, Lung neoplasms, Tumor markers, Diagnostic efficacy

## Abstract

**背景与目的:**

宣威肺癌近年来发病率持续增加，还具有全年龄段高发、女性肺癌死亡率高等特点。因此寻找更多稳定的生物标志物用于宣威肺癌的诊断具有巨大的临床应用前景。本研究旨在探究4个微小RNA（microRNA, miRNA）各自及联合传统肺癌肿瘤标志物在宣威肺癌检测和诊断中的临床应用价值。

**方法:**

通过检测4个miRNA和5种传统肺癌肿瘤标志物在45例宣威肺癌患者和健康体检者血清中的表达量，采用逻辑回归模型综合评估4个miRNA在宣威肺癌诊断中的灵敏度、特异度和诊断效能等相关统计学指标。

**结果:**

单个血清miRNA中miR-4646-5P、miR-3654在宣威肺癌组中的相对表达水平与对照组存在显著差异（P<0.05），miR-3654用于诊断的效果最好，灵敏度、特异度和曲线下面积分别为86.7%、82.2%、0.847。miR-3654联合miR-4646-5p、癌胚抗原（carcinoembryonic antigen, CEA）诊断宣威肺癌的效能最高，曲线下面积为0.901，灵敏度为73.3%，特异度为93.3%，阳性预测值为91.7%。

**结论:**

血清miR-3654在4个miRNA中对宣威肺癌诊断效果最好，并且联合miR-4646-5p、CEA能有效提高其对宣威肺癌的诊断价值，有望作为有价值的宣威肺癌筛查指标。

## Abstract (en)

**Background and objective:**

The incidence rate of lung cancer in Xuanwei has been continuously increasing in recent years, and it also features high incidence across all age groups and high mortality rates among female lung cancer patients. Therefore, the search for more stable biomarkers for the diagnosis of Xuanwei lung cancer holds tremendous clinical application prospects. This study aims to explore the clinical application value of these four microRNAs (miRNAs) individually and in combination with traditional lung cancer tumor markers in the detection and diagnosis of Xuanwei lung cancer.

**Methods:**

By detecting the expression levels of four miRNAs and five traditional lung cancer tumor markers in the serum of 45 Xuanwei lung cancer patients and healthy physical examination participants, the Logistic regression model was employed to comprehensively evaluate the sensitivity, specificity, diagnostic efficacy, and other relevant statistical indicators of the four miRNAs in the diagnosis of Xuanwei lung cancer.

**Results:**

Among the individual miRNAs, miR-4646-5p and miR-3654 showed significant differences in expression levels between the Xuanwei lung cancer group and the control group (P<0.05). miR-3654 demonstrated the best diagnostic performance with a sensitivity of 86.7%, specificity of 82.2%, and an area under the curve of 0.847. Combining miR-3654 with miR-4646-5p and carcinoembryonic antigen (CEA) resulted in the highest diagnostic efficacy for Xuanwei lung cancer, with a sensitivity of 73.3%, specificity of 93.3%, area under the curve of 0.901, and a positive predictive value of 91.7%.

**Conclusion:**

Among the four miRNAs, serum miR-3654 exhibits the best diagnostic efficacy for Xuanwei lung cancer. Combined with miR-4646-5p and CEA, its diagnostic value for Xuanwei lung cancer can be effectively enhanced, making it a promising screening indicator for Xuanwei lung cancer.

肺癌是全球范围内发病率和死亡率较高的恶性肿瘤之一，根据2020年全球数据^[1]^，肺癌死亡率居所有肿瘤的首位。中国是肺癌高发国，云南地区是中国肺癌的高发地区^[2]^，其中宣威地区是云南肺癌发病率和死亡率最高的地区之一^[3]^。宣威地区的肺癌患者发病率和死亡率在1973年后呈逐年上升趋势，且呈全年龄段高发^[4]^、女性肺癌死亡率高的特点，而且女性肺癌死亡率甚至可高达全国平均水平的7-9倍^[5]^。总体来看，肺癌给宣威市带来了较为严重的疾病负担^[6]^。目前对肺癌的初筛主要依赖于影像学和血清肿瘤标志物等方式检测^[7]^，因此从无创获取的血清样本中寻找更多可以用于初筛以及诊断宣威肺癌的稳定生物标志物，拥有巨大的临床应用前景^[8]^。目前研究人员已经发现许多肿瘤生物标志物^[9]^，这些标志物可以存在于癌组织和患者体液中，并且可以在成像和有创评估方法之前对肿瘤进行有效且快速的早期诊断^[10]^，并且联合使用多种标志物可以提高诊断准确率^[11]^，中华医学会肺癌临床诊疗指南（2023版）^[12]^、美国临床生化委员会、欧洲肺癌肿瘤标志物专家组均推荐5种肺肿瘤标志物：癌胚抗原（carcinoembryonic antigen, CEA）、神经元特异性烯醇化酶（neuron-specific enolase, NSE）、鳞状细胞癌相关抗原（squamous cell carcinoma associated antigen, SCC）、细胞角蛋白19片段（cytokeratin 19 fragment 21-1, CYFRA21-1）、前胃泌素释放肽（progastrin releasing peptide, ProGRP），虽然这些标志物不能取代组织学来确定病理类型，但是已被研究证明相对有效且被用于肿瘤诊断^[13]^。此外，微小RNA（microRNA, miRNA）作为液体活检的一类标志物，已被证明为可以应用于肺癌诊疗的潜在标志物^[14]^，miRNA在肺癌细胞增殖、分化和凋亡的过程中扮演了重要角色，通过调节多种信号传导通路影响肺癌细胞的生长、侵袭和耐药性等重要特性。例如miR-381-3P可介导HPV-16 E7癌蛋白进而诱导非小细胞肺癌血管生成^[15]^，miR-218-5p通过TPX2调节p53通路并抑制肺腺癌恶性进展^[16]^。此外，miR-103a-3p通过CYTOR/miR-103a-3p/HMGB1轴的调控，促进肺癌细胞增殖和转移等^[17]^。有研究^[18]^表明，将传统肺癌肿瘤标志物与miRNA结合使用，具有更高的临床应用价值。

基于此，本研究选择课题组在前期研究^[19]^中采用基因表达谱芯片筛选8对宣威肺腺癌患者癌组织样本及对应正常组织后，根据miRNA q-value(%)≤5，同时差异倍数（Fold Change）>2倍以上的标准从76个差异表达基因（http://www.lungca.org/files/2024s68-s1.pdf）中筛选出4个宣威肺癌中表达上调的miRNA：miR-3654、miR-3651、miR-720、miR-4646-5p，此4种miRNA在肺癌领域相关研究较少。miR-4646-5p相关研究主要集中在胃、乳腺癌^[20]^领域，目前仅有一项研究^[21]^涉及miR-4646-5p在肺癌中的作用。有研究^[22]^表明miR-3654的表达上调可导致多种肿瘤的发生和发展，目前miR-3654在肺癌方面的相关研究较少，Zhang等^[23]^发现miR-3654可与ceRNA网络中AL360219 和AC092071相互作用，可共同作为预测肺鳞状细胞癌预后的生物标志物。血清miR-3651被报道^[24]^可作为肺癌患者的诊断生物标志物。miR-720相关研究主要集中于乳腺癌领域^[25]^，有研究^[26]^发现，循环miR-720在不同癌症中表达水平存在较大不同，miR-720在非小细胞肺癌中的表达上调与不良临床表现及不良生存率有关，且表达水平会随时间出现差异。此4个miRNA与上述5种传统肺癌肿瘤标志物结合，采用逻辑回归模型验证和评估各项指标对宣威肺癌的诊断效能，结果通过接受者操作特征（receiver operating characteristic, ROC）曲线绘制，以ROC曲线下面积（area under the curve, AUC）等指标评估4个miRNA联合传统肺肿瘤标志物在宣威肺癌中的临床应用价值。

## 1 资料与方法

### 1.1 研究对象

收集2020年9月至2021年10月于昆明医科大学第一附属医院胸外科通过经皮肺穿刺活检或术后病理等诊断方式确认为肺腺癌^[27]^的45例宣威籍患者作为宣威肺癌组，宣威籍要求患者均于宣威地区居住超过15年且很少或从未在其他地区长期居住，取其手术前均未接受过放化疗及免疫治疗^[28]^的血清样本。另收集2020年9月至2021年10月于昆明医科大学第一附属医院健康体检中心体检的45例影像及实验室检测结果均在正常参考区间内且未合并其他免疫性疾病、消耗性疾病及恶性肿瘤^[29]^的健康体检者作为对照组。收集相应组别血清样本后进行两步离心（4 ^o^C、1500 g离心10 min，4^ o^C、13,000 g离心15 min）以消除细胞沉淀物，取其部分血清通过全自动化学发光法检测其传统肺癌肿瘤标志物表达水平，另一部分转移至1.5 mL的无RNase酶EP管中，于-196^ o^C液氮中保存，直至进行miRNA提取。本研究经相关医院的伦理委员会批准，患者均签署知情同意书。

### 1.2 统计效能与样本例数估计

检测血清中含量时采用2^-ΔΔCT^法检测宣威肺癌患者、健康人群血清中4个miRNA的相对定量，^Δ^Ct=（目的基因Ct-内参基因Ct），^ΔΔ^Ct=（宣威肺癌目的基因^Δ^Ct-健康人群目的基因^Δ^Ct），当log_2_a^-ΔΔCT^>1时定义为表达水平高，<-1定义为表达水平低，在两者之间定义为表达无改变。各组例数计算采用样本含量估算公式为n=2[（t_ɑ_+t_β_)s/δ]^2^，以Z界值表数据迭代2（n-1）自由度至稳定自由度数值，自由度向上取整，其中s为标准差，δ为容许误差，检验水准α概率为0.1，二类错误β概率为0.2，检验效能1-β=0.8，计算时按照公式要求取单侧概率。计算例数采用数据来源预实验时通过实时荧光定量聚合酶链式反应（quantitative polymerase chain reaction, qPCR）检测3例宣威肺癌组患者血清和3例对照组健康者中miRNA的表达水平。

#### 1.2.1 以miR-4646-5p含量为例

宣威肺癌组：s=2.654，均值=2.42，对照组：s=0.009，均值=0.21，δ=宣威肺癌组均值-对照组均值=2.42-0.21=2.21，s取2.654，查Z界值表得，单侧Z_0.05_=1.645、单侧Z_0.10_=1.282代入公式得，n_1_=2[（1.645+1.282）×2.654÷2.21]^2^≈25。以2n_1_-2=25×2-2=48作为自由度，查t界值表得，单侧t_0.05，48_=1.676，t_0.10，48_=1.299，代入公式得，n_2_=2[（1.676+1.299）×2.654÷2.21]^2^≈26，以2n_2_-2=52-2=50作为自由度，查t界值表得，单侧t_0.05，50_=1.676，t_0.10，50_=1.299，带入公式得，n_3_=2[（1.676+1.299）×2.654÷2.21]^2^≈26。因此，每组至少需要26个样本才能得到结果的差异有统计学意义的结论。

#### 1.2.2 以miR-3654含量为例

宣威肺癌组：s=51.55，均值=55.69，对照组：s=3.85，均值=2.24，δ=宣威肺癌组均值-对照组均值=55.69-2.24=53.45，s取51.55，查Z界值表得，单侧Z_0.05=_1.645、单侧Z_0.10_=1.282代入公式得，n_1_=2[（1.645+1.282）×51.55÷53.45]^2^≈16，以2n_1_-2=16×2-2=30作为自由度，查t界值表得，单侧t_0.05，30_=1.697，t_0.10，30_=1.310，代入公式得，n_2_=2[（1.697+1.310）×51.55÷53.45]^2^≈17，以2n_2_-2=34-2=32作为自由度，查t界值表得，单侧t_0.05，32_=1.694，t_0.10，32_=1.309，带入公式得，n_3_=2[（1.694+1.309）×51.55÷53.45]^2^≈17，因此，每组至少需要17个样本才能得到结果的差异有统计学意义的结论。

#### 1.2.3 以miR-3651含量为例

宣威肺癌组：s=2134.78，均值=1473.62，对照组：s=0.595，均值=0.345，δ=宣威肺癌组均值-对照组均值=1473.62-0.34=1473.28，s取1473.62，查Z界值表得，单侧Z_0.05_=1.645、单侧Z_0.10_=1.282代入公式得，n_1_=2[（1.645+1.282）×1473.62÷1473.27]^2^≈1，以2n_1_-2=2×2-2=2作为自由度，查t界值表得，单侧t_0.05，2_=2.92，t_0.10，2_=1.886，代入公式得，n_2_=2[（2.92+1.886）×1473.62÷1473.27]^2^≈1，因此，每组至少需要1个样本才能得到结果的差异有统计学意义的结论。

#### 1.2.4 以miR-720含量为例

宣威肺癌组：s=6.49，均值=4.76，对照组：s=1.730，均值=0.98，δ=宣威肺癌组均值-对照组均值=6.49-1.73=4.76，s取6.49，查Z界值表得，单侧Z_0.05_=1.645、单侧Z_0.10_=1.282代入公式得，n_1_=2[（1.645+1.282）×6.49÷4.76]^2^≈32，以2n_1_-2=32×2-2=62作为自由度，查t界值表得，单侧t_0.05，62_=1.667，t_0.10，62_=1.294，代入公式得，n_2_=2[（1.667+1.294）×6.49÷4.76]^2^≈33。以2n_2_-2=66-2=64作为自由度，查t界值表得，单侧t_0.05，64_=1.667，t_0.10，64_=1.294，带入公式得，n_3_=2[（1.667+1.294）×6.49÷4.76]^2^≈33。因此，每组至少需要33个样本才能得到结果的差异有统计学意义的结论。结合四组结果，在检验水准α概率为0.1、二类错误β概率为0.2的情况下，共需要33个以上样本才能得到统计效能在80%以上的结果。因此本研究采用45例样本以提高统计效能。

### 1.3 实验过程

采用miRcute miRNA Isolation Kit（qiagen）试剂盒提取目的血清中的miRNA。使用核酸/蛋白定量分光光度仪器（Eppendorf, German）测量miRNA的含量和纯度。使用miRcute Plus miRNA First-Strand cDNA Kit（qiagen）加尾法试剂盒进行miRNA逆转录以合成cDNA，使用核酸/蛋白定量分光光度仪器（Eppendorf, German）测量miRNA的含量和纯度。最后使用miRcute Plus miRNA qPCR Detection kit（qiagen）进行qPCR以检测miRNAs的表达水平。U6作为内源性对照。引物序列见表1。通过2^-ΔCT^比较方法分析qPCR结果，qPCR产物使用琼脂糖凝胶电泳评估完整性，图像是否清晰，位置是否正确，并配合相应的RT-qPCR熔解峰曲线判断有无特异度扩增或引物二聚体。

**表1:** 引物序列

Gene symbol	Primer sequence (5’-3’)
miR-4646-5p-F	GGCACTGGGAAGAGGAGCT
miR-3654-F	CTGGACAAGCTGAGGAA
miR-3651-F	CATAGCCCGGTCGCTGGT
miR-720-F	CCGGCTCTCGCTGGGG
U6-F	CTCGCTTCGGCAGCACA

F means forward primer, synthesis of cDNA by tail addition method, and the reverse primers were included in the miRcute Plus miRNA qPCR Detection kit.

### 1.4 患者miRNA、血清传统肺癌肿瘤标志物水平和其他资料分析与评估

收集并分析参与实验的宣威肺癌组、对照组中的患者年龄、性别等一般资料，检测并收集患者NSE、CEA、CYFRA21-1、ProGRP和SCC检测结果，分析miRNA的实验结果与这些临床资料的关联性，对存在关联的指标进行ROC曲线分析。

### 1.5 统计学分析

采用SPSS 24.0和MedCalc进行统计学分析处理，计量资料采用单样本Kolmogorov-Smirnov和Shapiro-Wilk检验进行正态性检验，对正态分布的计量资料用Mean±SD表示，两组间比较采用独立样本t检验，非正态分布的计量资料用中位数（P_25_, P_75_）表示，组间比较采用秩和检验。计数资料组间比较采用χ^2^检验，采用二元Logistic回归模型进行诊断分析。采用MedCalc生成ROC曲线、AUC、灵敏度、特异度、约登指数（Youden's index, YI）、似然比评价各指标与肺癌的关系，采用Graphpad和MedCalc进行绘图。本研究采用双侧检验，检验水准α=0.05作为统计检验水平，除了单样本Kolmogorov-Smirnov和Shapiro-Wilk检验要求P>0.05，其余统计学检验结果P<0.05为差异具有统计学意义。

## 2 结果

### 2.1 血清miR-4646-5p、miR-3654在宣威肺癌组与对照组中存在显著差异

对宣威肺癌组和对照组的4个血清miRNA含量进行检测和统计学检验，结果可见：4个miRNA均为非正态分布，宣威肺癌组miR-4646-5p、miR-3654相对表达水平均显著高于对照组（P<0.05）（图1，表2），miR-3651、miR-720相对表达水平高于对照组，但无显著差异（P>0.05）。

**图1:**
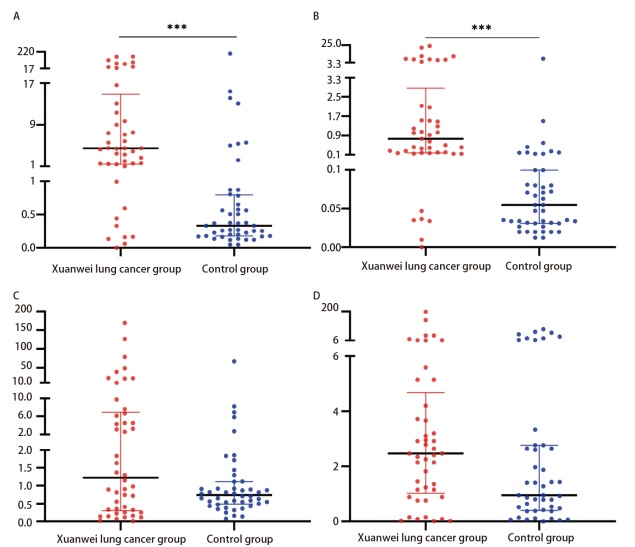
4种miRNA在宣威肺癌组与对照组的相对表达量。A：miR-4646-5p；B：miR-3654；C：miR-3651；D：miR-720。内线代表中位数，而底线和顶线分别代表P_25_和P_75_所在位置。***P<0.001。

**表2:** 4种miRNA在宣威肺癌组与对照组相对表达量及比较

miRNA	Xuanwei lung cancer group (n=45)	Control group (n=45)	Z	P
miR-4646-5p	4.507 (1.426, 14.847)	0.329 (0.175, 0.794)	4.697	0.001
miR-3654	0.763 (0.176, 2.867)	0.055 (0.031, 0.010)	5.678	0.001
miR-3651	1.145 (0.292, 6.392)	0.715 (0.488, 1.015)	1.380	0.168
miR-720	2.469 (1.025, 4.474)	0.958 (0.506, 2.768)	8.692	0.053

All miRNA results for each group were represented by the median (P_25_, P_75_).

### 2.2 血清传统肺癌肿瘤标志物水平在宣威肺癌组与对照组中存在显著差异

对宣威肺癌组和对照组的一般资料因素（年龄、性别、是否吸烟）进行统计学检验，结果显示以45岁为分界^[12]^的两组年龄存在统计学差异（P<0.05）（表3），性别和是否吸烟无统计学差异（P>0.05），此结果也与宣威肺癌在年龄段高发的特点相符^[4]^。对两组血清传统肺癌肿瘤标志物水平（CEA、NSE、SCC、CYFRA21-1、ProGRP）进行检测，结果显示5种标志物中除CEA、ProGRP外均为正态分布，宣威肺癌组CEA表达水平均显著高于对照组（P<0.05），其余组别无显著差异（P>0.05）（图2，表4）。

**表3:** 宣威肺癌组与对照组的一般资料分析

Influence factor		Xuanwei lung cancer group (n=45)		Control group (n=45)	χ^2^	P
Age (yr)	≥45	38		26	7.790	0.005
	<45	7		19		
Gender	Male	27		26	0.046	0.830
	Female	18		19		
Smoking status	Yes	26		27	0.046	0.830
	No	19		18		

**图2:**
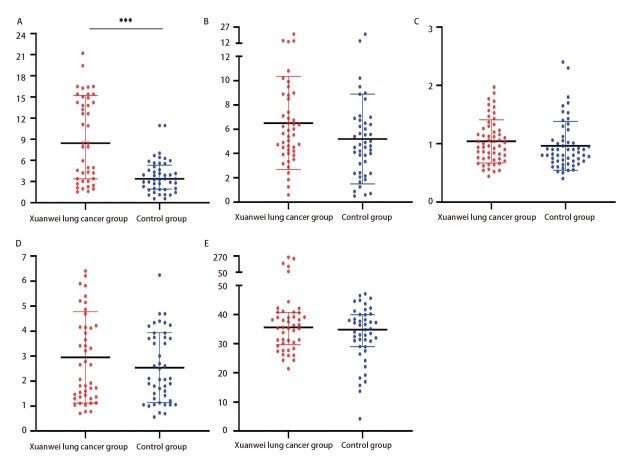
宣威肺癌组与对照组中传统肺癌肿瘤标志物含量比较。A：CEA；B：NSE；C：SCC；D：CYFRA21-1；E：ProGRP。正态分布数据内线代表标准差，底线和顶线代表标准差范围。非正态分布数据内线代表中位数，而底线和顶线分别代表P_25_和P_75_。***P<0.001。

**表4:** 宣威肺癌组与对照组中传统肺癌癌症肿瘤标志物含量比较

Tumor markers	Xuanwei lung cancer group (n=45)	Control group (n=45)	P
CEA	8.430 (3.375, 15.040)	3.360 (1.930, 5.315)	<0.001
NSE	6.509±3.835	5.195±3.694	0.101
SCC	0.968±0.410	0.952±0.327	0.837
CYFRA21-1	2.950±1.828	2.536±1.400	0.431
ProGRP	35.600 (29.650, 40.700)	34.80 (28.950, 39.950)	0.451

The results of traditional lung cancer tumor markers with normal distribution are shown as Mean±SD, while the results with non-normal distribution are represented by median (P_25_, P_75_).

### 2.3 miR-720联合CEA可作为有价值的宣威肺癌诊断指标

将4个miRNA和5种传统肺癌肿瘤标志物的二元Logstic回归模型预测结果均进行ROC曲线分析，其目的是在两组差异非显著假设的前提下，分析所有指标是否具有潜在的区分宣威肺癌和健康体检者的效能，这样可以避免遗漏并且全面展示4个miRNA在宣威肺癌诊断中的价值。

4个miRNA的ROC曲线结果分析得到：miR-3654的诊断效能最高，其灵敏度和特异度分别为86.7%和82.2%，AUC为0.847。miR-4646-5p的灵敏度和特异度分别为82.2%和82.2%，AUC为0.787。miR-3651的灵敏度和特异度分别为51.1%和80.0%，AUC为0.584。miR-720的灵敏度和特异度分别为62.2%和71.1%，AUC为0.618（图3，表5）。CEA在5种传统肺癌肿瘤标志物中具有最高的诊断效能，灵敏度和特异度分别为57.8%和95.6%，AUC为0.773（图4，表5）。

**图3:**
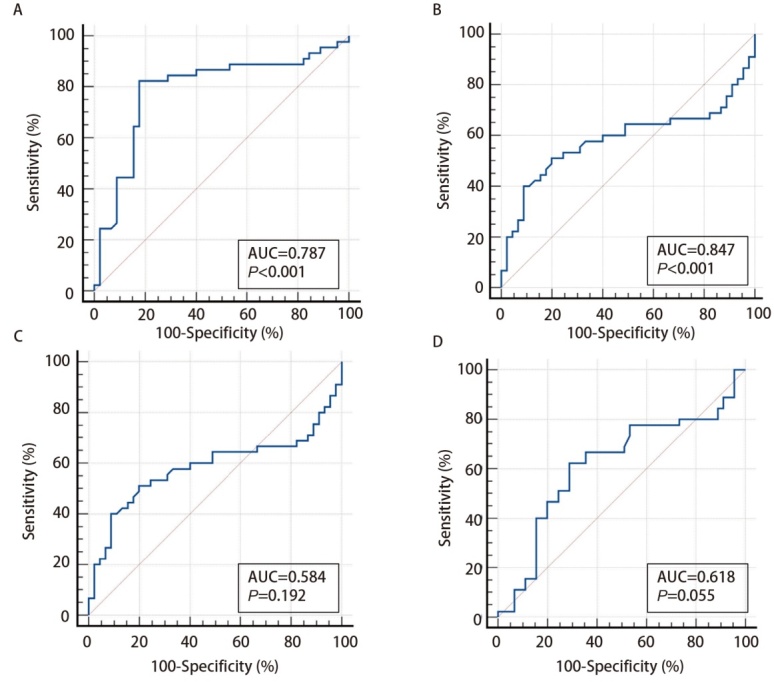
单个miRNA在宣威肺癌诊断中的ROC曲线结果。A：miR-4646-5p；B：miR-3654；C：miR-3651；D：miR-720。

**表5:** 单个miRNA和传统肺癌肿瘤物标志物在宣威肺癌诊断中的ROC曲线结果

Indicator name	ROC area	Sensitivity (%)	Specificity (%)	Youden’s index	95%CI	+LR	-LR	P
miR-4646-5p	0.787	82.2	82.2	0.644	0.689-0.867	4.62	0.22	<0.001
miR-3654	0.847	86.7	82.2	0.689	0.756-0.915	4.87	0.16	<0.001
miR-3651	0.584	51.1	80.0	0.311	0.476-0.687	2.56	0.61	0.192
miR-720	0.618	62.2	71.1	0.321	0.504-0.719	2.07	0.53	0.055
CEA	0.773	57.8	95.6	0.533	0.673-0.855	13.00	0.44	<0.001
NSE	0.614	93.3	24.4	0.178	0.506-0.714	1.24	0.27	0.054
SCC	0.518	62.2	46.7	0.090	0.410-0.624	1.17	0.81	0.776
CYFRA21-1	0.535	87.2	34.0	0.213	0.429-0.639	1.32	0.37	0.568
ProGRP	0.546	71.1	48.9	0.196	0.438-0.651	1.39	0.59	0.462

95%CI: 95% confidence interval; +LR: positive likelihood ratio; -LR: negative likelihood ratio.

**图4:**
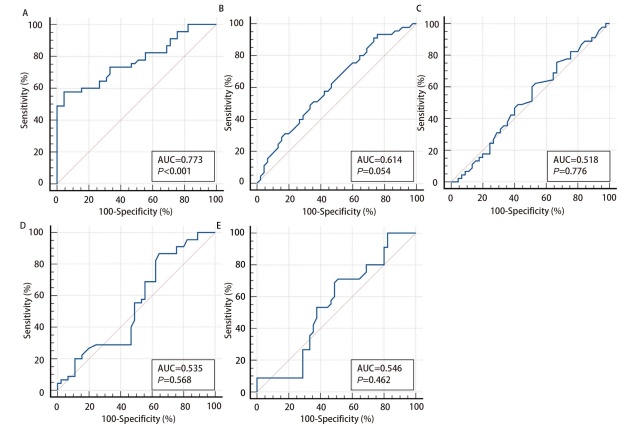
传统肺癌肿瘤物标志物在宣威肺癌诊断中的ROC曲线结果。A：CEA；B：NSE；C：SCC；D：CYFRA21-1；E：ProGRP。

为了进一步探究检验联合诊断指标是否能获得更好的诊断能力，将具有显著区分效能的miR-4646-5p、miR-3654、CEA分为两组，即miR联合组（miR-4646-5p+miR-3654）、总指标联合组（miR-4646-5p+miR-3654+CEA）。miR联合组的灵敏度为86.7%，特异度为80.0%，AUC为0.874，阳性预测值为81.3%。总指标联合组在所有指标中拥有最高的诊断效能，灵敏度为73.3%，特异度为93.3%，AUC为0.901，阳性预测值为91.7%（图5，表6），所有miRNA联合组的诊断效能高于单个miRNA。

**图5:**
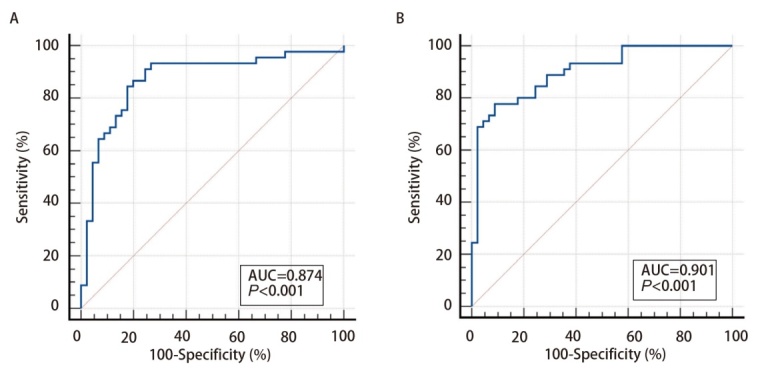
2个miRNA联合组别诊断宣威肺癌的ROC曲线结果。A：miR联合组；B：总指标联合组。

**表6:** 2种miRNA联合组诊断宣威肺癌的ROC曲线的各项数值

Indicator name	ROC area	Sensitivity (%)	Specificity (%)	Youden’s index	95%CI	+LR	-LR	PV+ (%)	PV- (%)
miRNAs combination group	0.874	86.7	80.0	0.667	0.787-0.934	4.33	0.17	81.3	99.9
All tumor marks combination group	0.901	73.3	93.3	0.689	0.820-0.954	8.75	0.24	91.7	99.9

The incidence rate of Xuanwei lung cancer used in calculating the positive and negative predictive value is 322.98/100,000^[6]^. PV+: positive predictive value; PV-: negative predictive value.

## 3 讨论

本研究实验数据选出可用的miR-4646-5p和miR-3654，通过miRPathDB（https://mpd.bioinf.uni-sb.de/）生成的富集热图（http://www.lungca.org/files/2024s68-s2.pdf）可以发现miR-4646-5p、miR-3654均显著富集集中于DNA结合转录因子活性、RNA特异性聚合酶II表达种类的通路上，miRWalk（http://mirwalk.umm.uni-heidelberg.de/）的GSEA富集通路显示其可以共同作用于蛋白质丝氨酸/苏氨酸激酶活性通路及其相关成纤维细胞生长因子受体1、肌醇六磷酸激酶活性通路、小GTP酶结合蛋白通路等781种通路（http://www.lungca.org/files/2024s68-s2.pdf），根据TargetScan（https://www.targetscan.org/）的预测，两种miRNA也可能共同作用于上述通路相应的成纤维细胞生长因子受体1、肌醇六磷酸激酶、细胞质FMR1相互作用蛋白1等6147种基因（http://www.lungca.org/files/2024s68-s3.pdf）。

对于本研究选取的4种miRNA中miR-4646-5p、miR-3654、miR-3651均无在肺癌中的研究论文发表，只有一位研究者^[30]^提到了miR-720在脑转移性肺腺癌中表达上调，提示miR-720可能在肺癌转移机制中发挥部分作用。本研究的结果也显示miR-720在宣威肺癌中表达升高。前期结果^[19]^显示，miR-4646-5p在肺癌中的表达量是正常人的2.46倍，miR-3654是132.07倍，miR-3651是4.68倍，miR-720是4.129倍，其Score分别为2.69、4.12、4.87和3.24。与类似的miRNA肺癌标志物研究结果进行比对，可发现上述2种miRNA对肺癌的诊断效能和同类研究中的单个miRNA类似（如：miR-9-5p，AUC=0.706；miR-21-5p，AUC=0.765；miR-223-3p，AUC=0.744^[31]^；miR-34b-3p联合miR-302a-5p，AUC=0.832^[32]^），而且本研究发现的总指标联合组（miR-3654联合miR-4646-5p、CEA）其诊断效能有较大的提高，血清标志物具有无创、获取简便、低成本等优点，因此总指标联合组在肺癌的诊断方面具有广阔的应用和研究前景，需要注意的是，对于本研究所涉及的肿瘤标志物，在应用时不仅需要考虑其具体表达量与健康人群是否存在显著差异，还需要考虑其是否在异常范围内，进行综合分析和评估其具体诊断情况^[33,34]^。本研究为宣威肺癌诊断和治疗提供了新的思路和方法，总指标联合组有望成为有价值的宣威肺癌筛查指标。

本研究也存在一些不足，纳入的所有组别研究对象均来自于昆明医科大学第一附属医院，后期需要收集更多中心的临床样本进行研究，本研究筛选的4个miRNA作为诊断辅助标志物的实用性在宣威肺癌诊断中尚未被报道。血清miRNA的来源复杂，目前尚无同种miRNA在组织或细胞中表达水平与血清中是否一致的相关研究。因此，可以进一步扩大样本数量和类型，检测组织或细胞中表达水平是否与血清中的miRNA一致，通过生信分析、查找相关数据库，探讨miRNA的作用和机制，并结合相关实验，以更全面、有效和深入地评估其在宣威肺癌及相关病症中的临床应用价值。
